# An Optimized Channel Selection Method Based on Multifrequency CSP-Rank for Motor Imagery-Based BCI System

**DOI:** 10.1155/2019/8068357

**Published:** 2019-05-13

**Authors:** Jian Kui Feng, Jing Jin, Ian Daly, Jiale Zhou, Yugang Niu, Xingyu Wang, Andrzej Cichocki

**Affiliations:** ^1^Key Laboratory of Advanced Control and Optimization for Chemical Processes, Ministry of Education, East China University of Science and Technology, Shanghai, China; ^2^Brain-Computer Interfacing and Neural Engineering Laboratory, School of Computer Science and Electronic Engineering, University of Essex, Colchester CO4 3SQ, UK; ^3^Skolkowo Institute of Science and Technology (SKOLTECH), 143026 Moscow, Russia; ^4^Systems Research Institute PAS, Warsaw, Poland; ^5^Nicolaus Copernicus University (UMK), Torun, Poland

## Abstract

**Background:**

Due to the redundant information contained in multichannel electroencephalogram (EEG) signals, the classification accuracy of brain-computer interface (BCI) systems may deteriorate to a large extent. Channel selection methods can help to remove task-independent electroencephalogram (EEG) signals and hence improve the performance of BCI systems. However, in different frequency bands, brain areas associated with motor imagery are not exactly the same, which will result in the inability of traditional channel selection methods to extract effective EEG features.

**New Method:**

To address the above problem, this paper proposes a novel method based on common spatial pattern- (CSP-) rank channel selection for multifrequency band EEG (CSP-R-MF). It combines the multiband signal decomposition filtering and the CSP-rank channel selection methods to select significant channels, and then linear discriminant analysis (LDA) was used to calculate the classification accuracy.

**Results:**

The results showed that our proposed CSP-R-MF method could significantly improve the average classification accuracy compared with the CSP-rank channel selection method.

## 1. Introduction

Brain-computer interface (BCI) technology enables the human brain to communicate directly with the outside world through electroencephalogram (EEG) signals and has attracted considerable attention in recent years [1]. BCIs can translate brain signals into output commands that allow users to control external auxiliary devices (such as wheelchairs, robotic arms, etc.) [2, 3]. Since BCIs provide an alternative way for people to communicate without use of peripheral nerves and muscles, they show a great value of, for instance, helping patients with severe neuromuscular disorders such as spinal cord injury or amyotrophic lateral sclerosis to restore their communication pathways and control their environment [4, 5].

Compared with evoked potential-based BCIs, motor imagery- (MI-) based BCIs have the advantages of being independent of external stimuli and easier to operate. MI-based BCI has been shown to be suitable for mechanical control and exercise rehabilitation training [6]. However, the brain signals used to control MI movements suffer from a number of problems including, but not limited to, low spatial resolution and low signal-to-noise ratios and are susceptible to strong artifacts [7, 8]. Researchers have used many feature extraction methods to address these difficulties, such as wavelet transforms, wavelet packet transforms, autoregressive (AR) models, and common spatial patterns (CSP). CSP, in particular, has been widely used for feature extraction to improve the performance of MI-based BCIs [9]. The use of multichannel signals tends to achieve good classification performance [10]. Nevertheless, multichannel signals typically carry a large amount of redundant information, which introduces additional noise sources and may decrease EEG-based motor imagery classification accuracy when compared to a small set of optimal EEG channels [11]. Channel selection can effectively exclude the redundant channels and select the optimal brain areas for MI-based BCIs. Therefore, channel selection is an important method of feature extraction for MI-based BCI [12].

In recent years, researchers have proposed many algorithms for channel selection, such as sequential floating forward selection (SFFS) [12], the mutual information-based channel selection method [13], support vector machine recursive feature elimination (SVM-rfe) [14], and CSP-rank [15, 16]. Among them, CSP-rank is one of the most frequently used channel selection methods [15, 16]. CSP-rank uses the projection matrix obtained by the CSP algorithm to sort and select the channels.

EEG is inherently noisy, and the signal-to-noise ratio is an important factor affecting the performance of BCIs [17, 18]. Many algorithms have been proposed to improve the signal-to-noise ratio of EEG. For example, the spatially sparsed common spatial pattern (SSCSP) method was proposed in [19], which has strong weights within the area of motor cortex and smooth weights elsewhere. The work in [20] proposed the spatially regularized common spatial pattern (SRCSP) method. Selim et al. [21] used root mean square (RMS) feature as inputs to an LDA classifier. Furthermore, Dai et al. [22] proposed the transfer kernel common spatial pattern (TKCSP) method to define the kernel of the domain-invariant by matching the division among source and target subjects. Park et al. [23] used noise-assisted multivariate extensions of empirical mode decomposition (NA-MEMD) to achieve a highly localized time-frequency representation. In [12], Qiu et al. proposed the improved sequential floating forward selection (SFFS) method, which combined the distribution of channels and an intelligent selection method (SFFS) to select channels for CSP in MI-based BCI. Finally, Feng et al. [8] designed a novel correlation-based time window selection (CTWS) algorithm for MI-based BCIs to address the time latency variation during an MI period between trials for each participant. These algorithms each worked in different ways to successfully improve the signal-to-noise ratio of EEG.

The cortical locations that are most heavily involved in motor control vary across EEG frequency bands [24]. Therefore, selecting channels in across different frequency bands could increase the discriminability of the extracted features further. However, traditional CSP-rank channel selection methods and the previous feature extraction algorithms did not consider the difference of channel configuration in different frequency bands [12, 15, 16] and could not suppress the interference from EEG features of different frequency bands, resulting in the degradation of their performance. In this paper, a new CSP-rank channel selection method is proposed, which considers the channel configuration in different frequency bands to increase the discriminability of the extracted features. The CSP-rank channel selection method was used to select the channels under a certain frequency band, and then the features are extracted by CSP using the selected channels. The extracted features from all frequency bands were concatenated to form one feature vector, which was improved further by the least absolute shrinkage and selection operator (LASSO). Linear discriminant analysis (LDA) was used as the classifier to demonstrate the performance of the method in terms of its impact on classification accuracy.

The paper is structured as follows: Section 2 describes the applied datasets and the proposed method.  Section 3 shows the evaluation results.  Section 4 presents the discussion about our method. Finally, concluding remarks are given in Section 5.

## 2. Methods

### 2.1. Description of the Data

**Figure 1:**
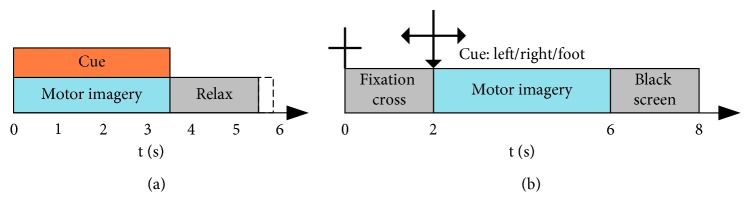
Illustration of the experimental protocol for a trial in dataset 1 (a) and dataset 2 (b). (b) is replaced from Feng et al. [8] (under the creative commons attribution license/public domain).

Dataset 1 (BCI Competition III datasets IVa): this dataset was recorded from 5 participants with 118 EEG channels. Visual cues were displayed for 3.5 s at a random interval uniformly drawn from the range 1.75 to 2.25 s. In the experiments, participants were instructed to perform three classes of MI movements: left hand, right hand, and right foot movements. Only data for the classes “right hand” and “right foot” were provided for the evaluation purpose. Each participant performed 140 trials for each class, respectively. A time window from 0 and 3.5 seconds, relative to cue presentation time, was used for feature extraction. The experiment process is illustrated in Figure 1(a). More details about the dataset can be found in the following website: http://www.bbci.de/competition/iii/desc_IVa.html.

Dataset 2 (BCI Competition IV dataset 1): the dataset was recorded from 7 participants with 59 EEG channels with a sampling rate of 100 Hz, including four healthy individuals and three artificially generated “participants.” For the purpose of the present study, only the calibration data (consisting of two runs totaling 200 trials) from the four healthy individuals were used. Each participant selected two classes of motor imagery from three available classes *left hand*, *right hand*, and *foot* motor imagery. Each trial started from a visual cue pointing left, right, or down. The cue was displayed for a period of 4 s, during which the participant was instructed to perform the cued motor imagery task. These periods were interleaved with 2 s of blank screen with a fixation cross shown in the center of the screen. The fixation cross was superimposed on the cues, i.e., it was shown for 6 s. Time window between 0.5 and 2.5 s was used for feature extraction. The experiment process is illustrated in Figure 1(b). More details can be found in the following website: http://www.bbci.de/competition/iv/desc_1.html.

### 2.2. CSP-Rank

CSP-rank was proposed based on the sorting of the CSP filter [13, 25]. CSP seeks a projection matrix to maximize the variance for one class and minimize the variance for another class at the same time in order to maximize the discriminability of the dataset across classes to aid classification. The CSP operation was achieved as follows:

(1) $$\begin{array}{c} \arg\;\;\max\frac{w^{\text{T}}C_{1}w}{w^{\text{T}}\left(C_{1}+C_{2}\right)w}, \end{array}$$

where *w* represented the projection vector and *C*_1_ and *C*_2_ represented the spatial covariance matrices of the two classes, respectively. We could regard it as the problem of finding generalized eigenvalues:

(2) $$\begin{array}{c} C_{1}w=\left(C_{1}+C_{2}\right)wD, \end{array}$$

where *D* denotes the diagonal matrix containing the eigenvalues of *C*_1_. We select eigenvectors SF1 and SF2 corresponding to the largest and smallest eigenvalues, respectively, from *w* as the projection matrix. These filter coefficients were used to assign different weights to different electrodes based on their importance. If the coefficient of a particular electrode was large, then that means the electrode was more important.

The original EEG data need to be filtered before selecting the channels. To achieve this, a 5th order Butterworth bandpass filter from 8 Hz to 30 Hz was used to filter EEG data [12]. The CSP-rank method first found the two CSP filters SF1 and SF2, then sorted the absolute value of the filter coefficients in SF1 and SF2, respectively, and took the electrode with the next largest coefficient in turn from the two spatial filters. If an electrode was already taken, then the procedure simply moved on to the next coefficient in the same spatial filter until a new electrode was reached. The search process did not stop until a stopping criterion was fulfilled. The stopping criterion selected was that accuracy no longer increased with the number of selected channels.

### 2.3. Channel Selection Method Based on CSP-Rank for Multiple Frequency Bands

**Figure 2:**
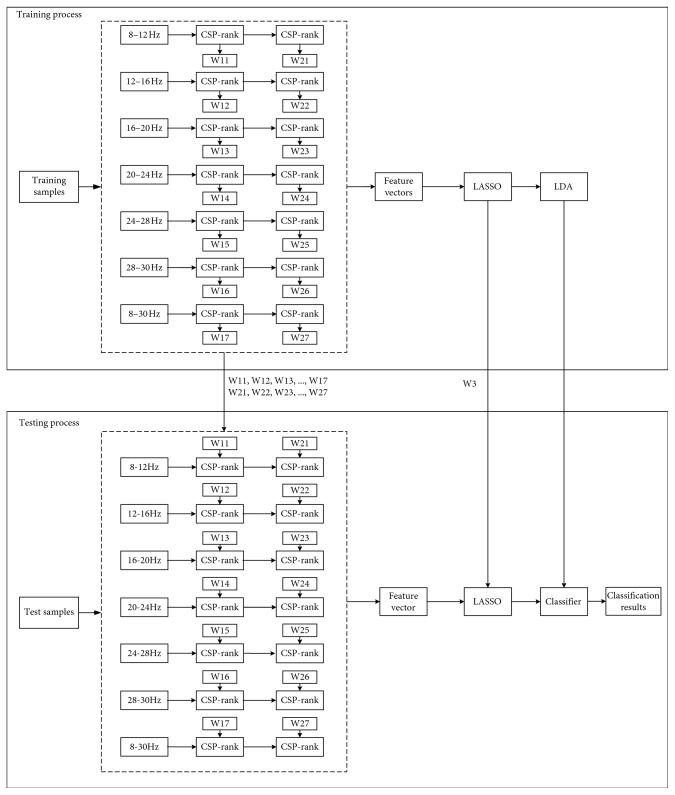
Structure diagram of the CSP-R-MF algorithm.

Figure 2 shows the structure diagram of the proposed method for optimizing EEG channel selection. Multiband signal decomposition filtering [25] was applied to the EEG signals recorded from all channels. Thereafter, the proposed CSP-rank method for reducing redundant channels was applied, and features were extracted with CSP from each frequency band. All features extracted from all frequency bands were concatenated to form one feature vector. The discriminant features were selected by LASSO from the feature vector. Finally, LDA was employed for the model training and accuracy calculation. The following subsections provide more details about the CSP-R-MF approach.

#### 2.3.1. Multiband Signal Decomposition Filter

A fixed time window was extracted from the trials of all the participants considered from the dataset. This time window began after the cue (beginning of motion imagination by the participants) and extended for 3.5 seconds for dataset 1 and 2 seconds for dataset 2 (the best window lengths for classification of dataset 2 were found to be among 1 s, 1.5 s, or 2 s [26].). Thereafter, seven frequency bands were considered covering the range 8–30 Hz. A fifth-order Butterworth filter was utilized to extract each band. The considered bandwidth was 4 Hz. Thus, the seven bands were defined as 8–12 Hz, 12–16 Hz, 16–20 Hz, 20–24 Hz, 24–28 Hz, 28–30 Hz, and 8–30 Hz.

For each subject, CSP-rank selection was used to remove several channels on each frequency band of the seven considered frequency bands. Thereafter, CSP was also applied on each frequency. Next, the CSP features were concatenated to form a high dimensional feature vector, which suffered from redundancies and irrelevant information. Such a feature vector might confuse the classifier. Therefore, an efficient feature selection algorithm was needed to select only the most relevant features.

#### 2.3.2. Least Absolute Shrinkage and Selection Operator

LASSO is a filter-based feature selection method and does not depend on any classifier; instead, they selected features according to statistical criteria. This selection method was on average more time efficient and more resistant to overfitting compared to wrapper-based feature selection methods [27]. It has also been shown to be efficient in feature selection for MI-based BCIs [28]. The goal of the algorithm was to minimize the residual sum of squared errors with a bound on the sum of absolute values of linear regression coefficients that had to be less than a given constant. The features would automatically be discarded corresponding to coefficients that were exactly 0. LASSO minimized the function:

(3) $$\begin{array}{c} \left(\frac{1}{2N}\overset{N}{\underset{i=1}{\sum }}{\left(y_{i}-\alpha_{0}-x_{i}^{\text{T}}\alpha \right)}^{2}+\lambda \overset{n}{\underset{j=1}{\sum }}\left|\alpha_{j}\right|\right), \end{array}$$

where *N* was the sample of samples, *y*_*i*_ was the response for sample *i*, *x*_*i*_ was the *n*-dimensional input vector for sample *i*, *λ* was a nonnegative regularization parameter, and *α*_0_ and *α* were regression parameters (*α*_0_ was a scalar; *α* was a *n*-dimensional vector). As *λ* increased, the number of nonzero components of *α* decreased. The values of parameters mentioned were set as default values in MATLAB R2015b built-in LASSO functions.

### 2.4. Classification Scheme

We used *n*-fold cross validation for performance validation. All samples were divided into *n* blocks for each fold cross validation. Nine blocks were used as training data, and the remaining one block was used as test data. Finally, the average classification accuracy of *n*-fold cross validation was selected as the estimation criteria. For dataset 1, each participant needs to perform 140 trials for each class; we selected 20 trials of 140 trials without repetition as the test data and the remaining trials as the training data for each cross validation. So, 7-fold cross validation was finally selected as the performance evaluation. However, each subject needed to perform 100 trials for each class in dataset 2; we selected 10 trails of 100 trials without repetition as the test data and the remaining trials as the training data for each cross validation. So, 10-fold cross validation was finally selected as the performance evaluation method. LDA was selected as the classifier. It classified samples by maximizing the distance between classes and minimizing intraclass variance and is often used in research of motor imagery-based BCI system [5].

### 2.5. Filter Bank Common Spatial Patterns

Filter bank common spatial pattern (FBCSP) was proposed by Kai et al. [29] to perform autonomous selection of key temporal spatial discriminative EEG characteristics. FBCSP comprises four progressive stages of EEG measurements processing: frequency filtering (the 5-fold Butterworth was selected in this paper), spatial filtering (CSP was selected in this paper), feature selection (LASSO was selected in this paper), and classification (LDA was selected in this paper). In the first stage, multiband signal decomposition filter was applied to the EEG signals. In the second stage, CSP was used to extract features of the EEG signals from each frequency band. In addition, all features extracted from all frequency bands were concatenated to form one feature vector. In the third stage, the discriminative parts of the feature vector were automatically selected with LASSO. In the fourth stage, LDA was selected to classify the discriminative parts of CSP features.

## 3. Results

**Figure 3:**
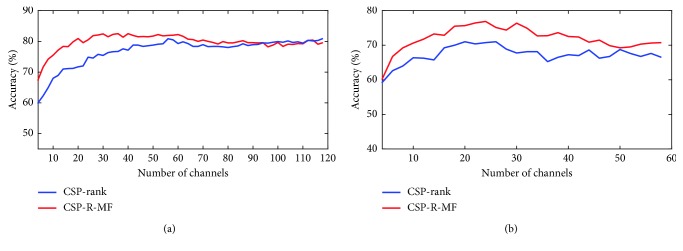
Overall accuracy curves showing the accuracy behavior with the varying numbers of channel in dataset 1 (a) and dataset 2 (b). The numbers of channels is the stopping criterion of the CSP-rank algorithm. The overall accuracy curves are the average over all subjects in each dataset. The red and blue lines denote the mean validation accuracy curves of two methods, respectively.

The overall accuracy behavior averaged from all the subjects in each dataset is shown in Figure 3. It may be observed that the CSP-R-MF achieves better performance in terms of classification accuracy. In the beginning, the classification accuracy increasingly corresponds to the increase in the number of selected channels. The classification accuracy would not increase further or would decrease a little, when more and more channels were selected. The peak point was 30 out of 118 channels for dataset 1, and the peak point was 24 out of 59 channels for dataset 2. So, we selected, respectively, 24 out of 59 channels for dataset 1 and 30 out of 118 channels for dataset 2 for later EEG analysis in each frequency band. Group level statistics are not reported as we do not have sufficient participants in this paper.

**Figure 4:**
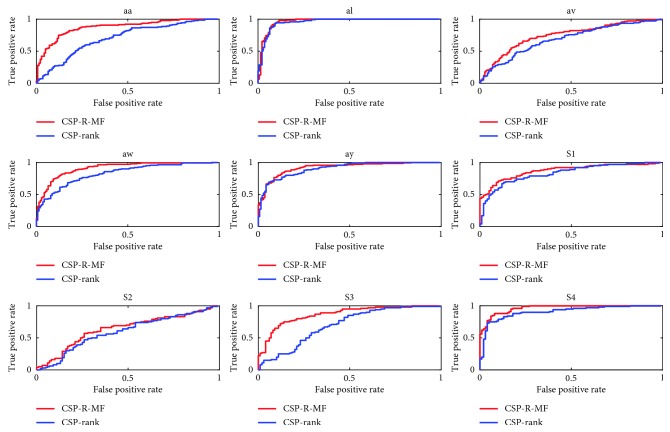
ROC curves of two methods for dataset 1 and dataset 2.

Figure 4 shows ROC curves for the two methods. The red line represents the accuracy achieved by the CSP-R-MF method proposed in this paper, and the blue line represents accuracy achieved by the CSP-rank method. Obviously, compared with the blue line, the red line generally tends to be in the upper left corner of the graph. This means that the model obtained by the method proposed in this paper has better performance than the model obtained by CSP-rank.

**Figure 5:**
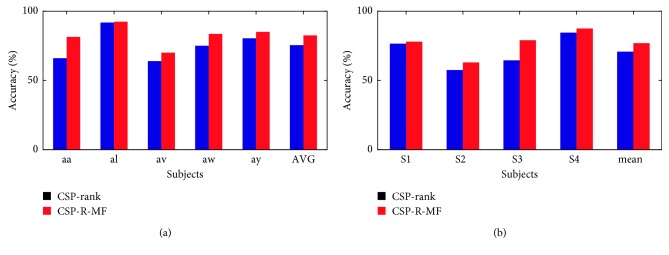
Performance comparison of the proposed algorithm CSP-R-MF with CSP-rank in dataset 1 (a) and dataset 2 (b).

Figure 5 shows the classification accuracies achieved with each of the two methods. The horizontal axis indicates the subject, and the vertical axis indicates the classification accuracy. The red bar represents CSP-R-MF results, and the blue bar represents CSP-rank results. Compared to CSP-rank, the classification accuracy of CSP-R-MF improved by 7.05% (75.43% VS 82.48%) for dataset 1 and 7% (70.75% VS 77.75%) for dataset 2. *P* value expresses the significance level. The smaller the *P* value, the higher the significance. In dataset 1, compared to CSP-rank, the classification accuracy of subjects aa, al, av, aw, and ay was, respectively, improved by 15.36%, 0.62%, 6.07%, 8.57%, and 4.64% with CSP-R-MF. In dataset 2, compared to CSP-rank, the classification accuracy of subjects S1, S2, S3, and S4 was, respectively, improved by 5%, 5.5%, 14.5%, and 3% with CSP-R-MF.

We also compared the performance of CSP-R-MF and FBCSP. The comparison results are shown in Table 1. Compared to FBCSP, the classification accuracy of CSP-R-MF improved by 2.91% for dataset 1 and 7% for dataset 2.  Table 2 shows the correspondence between electrodes and numbers.

## 4. Discussion

It is well established that motor imagery produces an event-related de\synchronization (ERD\S) over the sensorimotor areas within the mu rhythm (8–13 Hz) and the beta band (13–30 Hz) [7]. Therefore, the bandpass filter used for measuring the ERD/S was between 8 to 30 Hz [8, 12]. This frequency band may be further divided into 7 subbands (4–8 Hz, 8–12 Hz, 12–16 Hz, 16–20 Hz, 20–24 Hz, 24–28 Hz, 28–32 Hz, 32–36 Hz, and 36–40 Hz) to study the effect of frequency band selection on motor imagery-based BCI control, as proposed in work on subband common spatial pattern (SBCSP) [30] and FBCSP. It was found that the performance of the MI-based BCI correlated with these frequency bands. However, the best frequency band was not exactly the same for all subjects. In this paper, we selected 7 bands (8–12 Hz, 12–16 Hz, 16–20 Hz, 20–24 Hz, 24–28 Hz, 28–30 Hz, and 8–30 Hz) to study movement-related brain activity during motor imagery in different frequency bands.

Feature extraction is one of the most important steps for the classification of motor imagery EEG [31]. In particular, CSP, a spatial feature extraction method, has become the most commonly used method in MI-based BCI systems [12, 32]. However, the performance of the CSP is susceptible to interference signals. The channel selection algorithm selects the channels associated with motor imagery and removes the channels that do not contribute significantly to classification of motor imagery activity. This improves the signal-to-noise ratio of the EEG signals. Brain areas associated with motor imagery are not exactly the same in different frequency bands [17]. Therefore, the channels selected will not be the same in different frequency bands.

**Figure 6:**
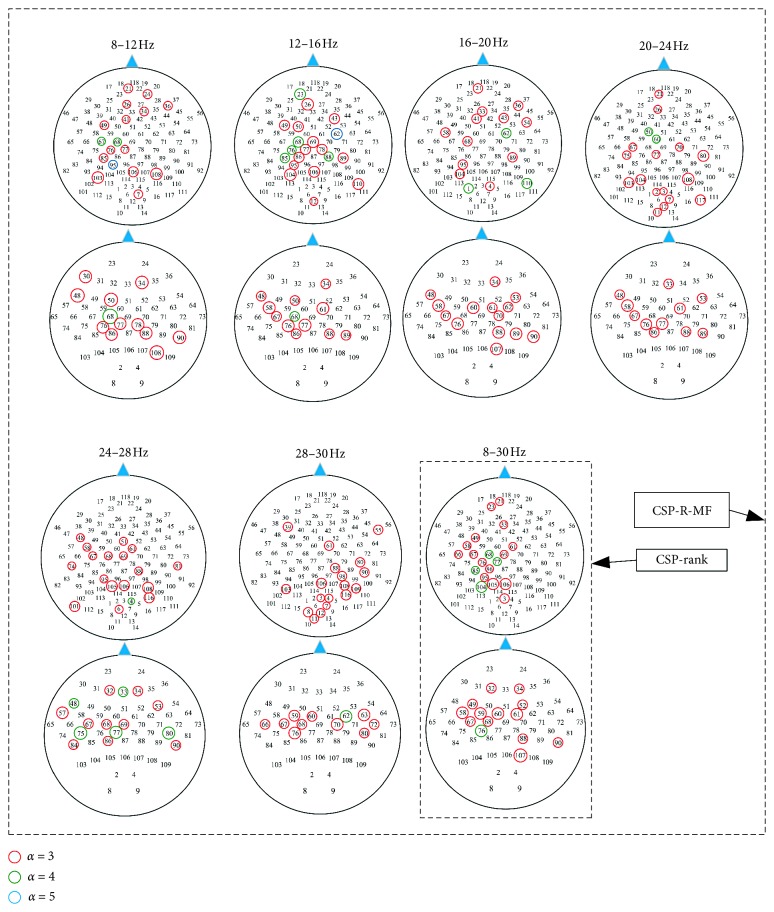
The topographic maps of the selected channels for two datasets under different frequency bands. Channels selected more than three times out of all subjects were marked. The channels selected by CSP-R-MF algorithm in 8–30 Hz frequency band were the same as those selected by CSP-rank algorithm. The electrode could be found in Table 2 with the number in topographic maps.

In this study, the proposed CSP-R-MF algorithm considered variations of brain areas involved in motor imagery in different frequency bands and automatically selected channels for each frequency band via the CSP-rank method.  Figure 6 shows the topographic maps of channels selected by CSP-R-MF in different frequency bands. The selected channels were marked with different colors according to the number of times each channel was selected.

As shown in Figure 6, the distributions of selected channels were different under different frequency bands. However, they were basically distributed in the motor areas of the cerebral cortex. This reflects the frequently observed fact that motor imagery involves EEG activity in relevant areas of the motor cortex [31, 33]. Channels selected under a few frequency bands were not mainly distributed in the motion areas. For example, the selected channels in the 16–20 Hz and 28–30 Hz bands were not mainly distributed in the motor areas for dataset 1, However, most of the selected channels under these two bands for dataset 2 were located in the motor area.

The ROC curve is a tool for evaluating the generalization performance of models. We compared the performance of two models based on CSP-rank and CSP-R-MF, respectively. Figures 3 and 5 showed that the performance of the model obtained by CSP-R-MF was better than the model obtained by CSP-rank. Figure 3 shows the accuracy behavior of CSP-R-MF and CSP-rank with the varying numbers of channel. The classification accuracy would increase corresponding to the increase in the number of selected channels in the beginning. The classification accuracy would decrease when more and more channels were selected. It proved the accuracy of the model has a globally optimal global region instead of falling into local optimum because of overfitting.

## 5. Conclusion

The brain areas associated with motor imagery are not exactly the same in different frequency bands [17]. Current state-of-the-art channel selection methods do not consider this problem. In this study, we proposed a novel method based on common spatial pattern- (CSP-) rank channel selection method for multifrequency band (CSP-R-MF) selection. In our approach, a 5th order Butterworth filter was used to achieve multiband signal decomposition filtering. After that, CSP-rank and CSP were used to extract features of filtered EEG samples. The discriminant characteristics were selected with LASSO. Finally, classification algorithms were employed to calculate the classification accuracy. Experimental results showed that the CSP-R-MF algorithm could improve performance of MI-based BCIs compared to the CSP-rank algorithm. More specifically, the average classification accuracy improved 7.05% on dataset 1 (BCI Competition III datasets IVa) and 7% on dataset 2 (BCI Competition IV dataset 1), when using the proposed method compared to using CSP-rank. In this paper, we set the same stopping criterion for all searching processes based on CSP-rank. However, the stopping criterion may be not exactly the same for different frequency bands and subjects. In future work, to further improve the performance of the CSP-R-MF algorithm, we will consider setting different stopping criteria for different frequency bands and subjects.

## Figures and Tables

**Table 1:** Classification accuracy of the proposed approach and FBCSP.

Dataset 1			Dataset 2		
Subject	FBCSP	CSP-R-MF	Subject	FBCSP	CSP-R-MF
aa	79.64	81.43			
al	96.79	92.41	S1	75	81.5
av	51.79	70	S2	54	63
aw	90.71	83.57	S3	61.5	79
ay	78.93	85	S4	92.5	87.5
Average	79.57	82.48	Average	70.75	77.75

**Table 2:** Correspondence table between electrodes and numbers.

Mark : electrode									
1 : PO3	2 : PO1	3 : POZ	4 : PO2	5 : PO4	6 : OPO1	7 : OPO2	8 : O1	9 : O2	10 : l1
11 : Ol1	12 : OZ	13 : Ol2	14 : l2	15 : PO7	16 : PO8	17 : AF7	18 : Fp1	19 : Fp2	20 : AF8
21 : AFP1	22 : AFP2	23 : AF3	24 : AF4	25 : FAF5	26 : FAF1	27 : FAF2	28 : FAF6	29 : F7	30 : F5
31 : F3	32 : F1	33 : Fz	34 : F2	35 : F4	36 : F6	37 : F8	38 : FFC7	39 : FFC5	40 : FFC3
41 : FFC1	42 : FFC2	43 : FFC4	44 : FFC6	45 : FFC8	46 : FT9	47 : FT7	48 : FC5	49 : FC3	50 : FC1
51 : FCz	52 : FC2	53 : FC4	54 : FC6	55 : FT8	56 : FT10	57 : CFC7	58 : CFC5	59 : CFC3	60 : CFC1
61 : CFC2	62 : CFC4	63 : CFC8	64 : CFC8	65 : T7	66 : C5	67 : C3	68 : C1	69 : Cz	70 : C2
71 : C4	72 : C6	73 : T8	74 : CCP7	75 : CCP5	76 : CCP3	77 : CCP1	78 : CCP2	79 : CCP4	80 : CCP6
81 : CCP8	82 : TP9	83 : TP7	84 : CP5	85 : CP3	86 : CP1	87 : CPz	88 : CP2	89 : CP4	90 : CP6
91 : TP8	92 : TP10	93 : PCP7	94 : PCP5	95 : PCP3	96 : PCP1	97 : PCP2	98 : PCP4	99 : PCP6	100 : PCP8
101 : P9	102 : P7	103 : P5	104 : P3	105 : P1	106 : Pz	107 : P2	108 : P4	109 : P6	110 : P8
111 : P10	112 : PPO1	113 : PPO5	114 : PPO1	115 : PPO2	116 : PPO6	117 : PPO8	118 : FPz	—	

Mark: electrode denotes electrode and its corresponding number. Mark: the number corresponding to the electrode.

## Data Availability

The data used to support the findings of this study are included within the article.
